# Differences in HDL Remodeling during Healthy Pregnancy and Pregnancy with Cardiometabolic Complications

**DOI:** 10.3390/antiox13080948

**Published:** 2024-08-03

**Authors:** Marko Stankovic, Aleksandra Zeljkovic, Jelena Vekic, Tamara Antonic, Daniela Ardalic, Milica Miljkovic-Trailovic, Jelena Munjas, Marija Saric Matutinovic, Tamara Gojkovic, Snezana Jovicic, Zeljko Mikovic, Aleksandra Stefanovic

**Affiliations:** 1 Gynecology and Obstetrics Clinic Narodni Front, 11000 Belgrade, Serbia; drmare84@hotmail.com (M.S.); danielaardalic@gmail.com (D.A.); mikovic.zeljko@gmail.com (Z.M.); 2 Department of Medical Biochemistry, Faculty of Pharmacy, University of Belgrade, 11000 Belgrade, Serbia; aleksandra.zeljkovic@pharmacy.bg.ac.rs (A.Z.); jelena.vekic@pharmacy.bg.ac.rs (J.V.); tamara.anotnic@pharmacy.bg.ac.rs (T.A.); milica.miljkovic@pharmacy.bg.ac.rs (M.M.-T.); jelena.munjas@pharmacy.bg.ac.rs (J.M.); marija.saric@pharmacy.bg.ac.rs (M.S.M.); tamara.gojkovic@pharmacy.bg.ac.rs (T.G.); snezana.jovicic@pharmacy.bg.ac.rs (S.J.); 3 Department of Gynecology and Obstetrics, Faculty of Medicine, University of Belgrade, 11000 Belgrade, Serbia

**Keywords:** pregnancy, gestational diabetes, hypertensive disorders of pregnancy, sphingosine-1-phosphate, paraoxonase 1, serum amyloid A, apolipoprotein A1

## Abstract

This study investigated the longitudinal trajectory of changes in antioxidative and anti-inflammatory high-density lipoprotein (HDL) components during healthy pregnancy and pregnancy with cardiometabolic complications. We recruited and longitudinally followed 84 women with healthy pregnancies and 46 pregnant women who developed cardiometabolic pregnancy complications (gestational diabetes mellitus and hypertensive disorders of pregnancy). Their general lipid profiles, oxidative stress status, inflammatory status, and antioxidative and anti-inflammatory HDL components were analyzed. The results of our study confirmed the expected trajectory for the routine lipid parameters. Our study results indicate more intensive oxidative stress and a higher level of inflammation in the group with complications compared with the control group. Sphingosine-1-phosphate (S1P) was significantly lower in the first trimester in the group with complications compared with the control group (*p* < 0.05). We did not find significant differences in the apolipoprotein A1 (Apo A1) concentrations in the first trimester between the control group and the group with complications, but in the second and third trimesters, the group with complications had significantly higher concentrations (*p* < 0.001, *p* < 0.05, respectively). The S1P, paraoxonase 1 (PON1), and serum amyloid A (SAA) concentrations were significantly lower in the group with complications in the first trimester. During the second trimester, only the SAA concentrations were identified as significantly lower in the group with complications compared with the control group, while in the third trimester, the PON1, apolipoprotein M (Apo M), and SAA concentrations were all significantly lower in the group with complications. Through a multivariate binary logistic regression analysis, the S1P concentration in the first trimester was distinguished as an HDL-associated marker independently associated with cardiometabolic pregnancy complications. In conclusion, our study results showed that HDL remodeling differs between healthy pregnancies and pregnancies with maternal cardiometabolic complications, with changed HDL composition and functionality consequently impacting its biological functionality in the latter case.

## 1. Introduction

Within healthy pregnancy, the adaptation of the mother’s metabolism to meet fetal needs for adequate development is an essential aspect. Hormones produced by the placenta (estrogen, progesterone, and human placental lactogen), as well as the significant increase in insulin secretion caused by pancreatic β-cell hyperplasia, play a crucial role in this process and regulate fetal and maternal metabolism [1]. Physiological changes in lipid metabolism during pregnancy are represented by an increase in serum levels of total cholesterol (TC) and triglycerides (TGs), followed by a moderate increase in low-density lipoprotein cholesterol (LDL-C) concentrations. The most intriguing aspect of maternal dyslipidemia that differentiates it from dyslipidemia in cardiometabolic diseases is the increase in the high-density lipoprotein cholesterol (HDL-C) concentration mid-gestation, which then tends to slightly decrease toward the end of pregnancy [2]. Excessive and abnormal changes in maternal lipid metabolism are profoundly associated with the development of pregnancy complications, especially with the most common maternal cardiometabolic conditions/disorders: gestational diabetes and hypertensive disorders of pregnancy [3,4]. Although these pregnancy complications have complex underlying mechanisms, they are closely related to disorders of maternal lipid metabolism and share several common risk factors (maternal age, pre-pregnancy obesity, and gestational weight gain) [3]. Despite intensive study of lipid profile alterations in gestational diabetes and hypertensive disorders of pregnancy in recent decades, the full scope and implications of dyslipidemia, especially changes in the metabolism, structure, and functionality of HDL, are still incompletely characterized [5,6,7].

An increase in HDL-C concentrations toward the end of the first trimester and during the second trimester of a physiological pregnancy is important for fetal cholesterol supply and is considered a protective metabolic adaptation associated with the stability of the maternal endothelium and favorable pregnancy outcomes [8,9,10,11]. However, we now understand that the role of HDL particles extends beyond their cholesterol content and therefore require thorough insight into HDL’s metabolism and structural and functional characteristics for a comprehensive understanding of its antiatherogenic properties [12,13]. Circulating HDLs consist of many different protein and lipid compounds and are concomitantly diverse, with distinct functional profiles [12]. Reverse cholesterol transport and the antioxidative, anti-inflammatory, antiapoptotic, anti-adhesive, and antithrombotic potential of HDL particles synergistically advance their ability to protect and improve endothelial function and contribute to their atheroprotective role [14]. In addition, while physiological pregnancy is associated with increased oxidative stress and mild inflammation, these conditions are intensified in cardiometabolic disturbances of pregnancy, such as gestational diabetes and hypertensive pregnancy disorders [15].

Other lipid species within HDL, especially sphingosine-1-phosphate (S1P), are important indicators of its functionality. S1P primarily interacts with HDL through its interaction with apolipoprotein M (Apo M), ensuring endothelium protection and maintaining vascular integrity by producing nitric oxide [16]. In the context of pregnancy, it has been shown that the HDL-apoM-S1P complex is paramount to maternal vascular protection and maintaining feto-placental endothelial function [17,18]. Further, the protein ensemble of HDL is very complex and prone to comprehensive structural and functional alterations in response to altered metabolic conditions. Apolipoprotein A1 (Apo A1), the major protein component of HDL, is integral to the architecture and function of HDL, acting as a primary mediator of cholesterol efflux and a very potent antioxidative tool under normal physiological conditions [19]. Given that previous results regarding the monitoring of Apo A1 during pregnancy and cardiometabolic pregnancy complications are inconsistent, additional research is necessary to fully grasp the significance of its changes and their patterns during gestation [20,21]. Further, in specific prolonged inflammatory conditions, liver-derived serum amyloid A (SAA) replaces ApoA 1 and other HDL proteins, which modifies HDL functionality [22]. Our current knowledge of this metabolic alteration is indicative of the need for a deeper understanding of the factors that modulate SAA’s association with HDL. Meanwhile, another antioxidant component associated with HDL is the enzyme paraoxonase (PON1), which can modulate signaling pathways that contribute to inflammation and oxidative stress [23]. Previous research has generally shown that PON1 levels and activity decrease in pregnancies with complications when compared to healthy pregnancies, but the exact clinical significance of this HDL modulation has not been entirely explained [20].

It is now evident that an unfavorable microenvironment can significantly affect HDL composition, its metabolism, and, as a consequence, its biological functionality [13]. In this paper, we will present the longitudinal trajectory of changes in antioxidative and anti-inflammatory HDL components that may affect their functionality during healthy pregnancy and in the context of cardiometabolic pregnancy complications, gestational diabetes, and hypertensive pregnancy disorders. Additionally, this study aimed to discern the most prominent characteristics in the HDL profile at the very beginning of pregnancy connected to the development of cardiometabolic pregnancy complications.

## 2. Materials and Methods

### 2.1. Subjects

This longitudinal study was part of a larger research project (HIgh-density lipoprotein MetabolOMe research to improve pregnancy outcome—HI-MOM) involving two cohorts of pregnant women recruited during their regular gynecological checkups at the Gynecology and Obstetrics Clinic “Narodni Front” in Belgrade, Serbia. The first cohort enrolled 131 pregnant women who were not at risk of pregnancy complication development. The exclusion criteria at the enrollment were the presence of any chronic disease before pregnancy, a body mass index (BMI) >30 kg/m^2^ before pregnancy, multiple gestation, molar pregnancy, or the intake of medication affecting their lipid profile. The recruitment of pregnant women into the second cohort was based on their pre-pregnancy risk of preeclampsia development in accordance with the guidelines established by the National Institute for Health and Care Excellence [24]. According to these recommendations, high- and moderate-risk conditions for preeclampsia development are delineated. High-risk factors include a high uterine artery pulsatility index (UtA Pi), chronic hypertension, chronic kidney disease, hypertension in previous pregnancy, diabetes mellitus type 1 or type 2, and autoimmune diseases, e.g., systemic lupus erythematosus, antiphospholipid syndrome, or a history of thrombophilia. Moderate-risk factors for the development of preeclampsia are a maternal age of 40 or older, first pregnancy, a pregnancy interval >10 years, and a family history of preeclampsia. The exclusion criteria for this cohort were multiple gestations, BMI >30 kg/m^2^ before pregnancy, the development of any infectious disease or the exacerbation of an existing autoimmune disease at any point during the follow-up, and treatment with lipid-lowering drugs. Following the application of the exclusion criteria, the second cohort included 90 pregnant women who were monitored until delivery. For both cohorts, their full medical history was taken, including noting the presence of any systemic disorders before pregnancy, smoking status, alcohol intake, vitamin supplementation, and family history of cardiovascular disease and diabetes mellitus. The study participants all reported following diets typical of their cultural context.

Although none of the study participants were professionally involved in sports, they all reported being moderately physically active. The study protocol also included height and weight measurements for BMI calculation, complete biochemical and hematology laboratory evaluation, and ultrasonographic and color Doppler diagnostics. Their arterial blood pressure was measured according to a standardized procedure, and their mean arterial pressure (MAP) was calculated [25]. Follow-up of the participants covered the entirety of pregnancy up until delivery. Complete clinical and laboratory evaluations were conducted toward the end of each trimester [1st trimester (median: 13.2 gestational week), 2nd trimester (median: 22.3 gestational week), and 3rd trimester (median: 32.9 gestational week)].

In the first cohort, 84 out of 131 pregnant women carried their pregnancies to term without any complications and served as the control group. In the second cohort, 47 delivered their babies without complications despite being at risk, but they were excluded from this research given that existing pre-pregnancy risk factors could have affected the parameters of interest. Of both groups of pregnant women, 46 women developed cardiometabolic pregnancy complications [13 gestational diabetes; 15 gestational hypertension, 12 preeclampsia; 2 gestational diabetes and gestational hypertension; and 4 gestational diabetes and preeclampsia] and served as the group of participants with complications.

All the study participants gave informed consent to participate in the study protocol, which was established in accordance with the Declaration of Helsinki. This research was approved by the Ethics Committee of Gynecology and Obstetrics Clinic “Narodni Front” (reference No. 05006-2020-10738); the Ethics Commission of the Faculty of Medicine, the University of Belgrade (designation No. 1322/VII-27); and the Ethical Committee for Biomedical Research of the Faculty of Pharmacy, the University of Belgrade (reference No. 1156/2).

### 2.2. Sampling and Methods

After fasting overnight (10 h), venous blood was extracted from the antecubital vein of each participant into a serum sample tube and an EDTA sample tube before immediate centrifugation at 1500× *g* for 10 min at 4 °C. The plasma and serum samples were kept in aliquots at −80 °C prior to analysis. Glucose and high-sensitivity C-reactive protein (hsCRP) concentrations and lipid profile parameters [TG, TC, HDL-C, Apo A1] were determined using an AU480 Beckman autoanalyzer employing routine commercial kits (Beckman, Brea, CA, USA). As the TG concentrations recorded were almost exclusively lower than 4.5 mmol/L, LDL-C levels were calculated using the Friedwald equation [26]. For the samples that did exhibit a concentration of TGs higher than 4.5 mmol/L, the LDL-C concentrations were determined using the AU480 Beckman autoanalyzer, employing commercial kits (Beckman, Brea, CA, USA).

Total antioxidant capacity (TAC) was determined using a colorimetric, automated method based on the discoloration of 2.2′-azinobis-(3-ethylbenzothiazoline-6-sulphonic acid) (ABTS) radical cations by serum antioxidants [27]. The intensity of the color change is proportional to the level of serum antioxidants. This method was calibrated with Trolox (a water-soluble analogue of vitamin E, 6-hydroxy-2.5.7.8-tetramethylchroman-2-carboxylic acid) and validated in our laboratory (with intra-assay and inter-assay coefficients of variance of 4.2% and 8.7%, respectively). Total oxidative status (TOS) was determined according to a similar methodological principle to that of the previous method, which was postulated by the same authors [28]. This method relies on the oxidation of ferrous iron into ferric iron in the presence of serum pro-oxidant components. Its intra-assay and inter-assay coefficients of variance are 5.6% and 9.5%, respectively. Alamdari’s method was used to measure the pro-oxidant antioxidant balance (PAB) as a marker of oxidative status [29]. Hydrogen peroxide and other oxidants (most often uric acid) concurrently react with 3,3′,5,5′-tetramethyl benzidine to yield a colored reaction product, which can be measured spectrophotometrically. The PAB levels are presented as special arbitrary HK units. We used the spectrophotometric method previously described by Richter and Furlong, with modifications within our laboratory, to determine PON1 activity [30].

Monocyte chemoattractant protein-1 (MCP-1) concentrations were determined using a Human MCP-1 ELISA Kit commercial sandwich immunoassay test (Wuhan Fine Biotech Co, Wuhan, China). Its intra-assay and inter-assay coefficients of variance were 7.9% and 12.5%, respectively. PON1 concentrations were measured using the commercial solid-phase sandwich Human Total PON1 DuoSet IC ELISA kit (DY1990, R&D Systems, Abingdon, UK/Minneapolis, MN, USA). Its intra-assay and inter-assay coefficients of variance were 5.3% and 8.1%, respectively. The Apo M concentrations were measured using a commercial ELISA (FineTest Biotech Inc., Wuhan, Hubei, China), with intra-assay and inter-assay coefficients of variance of 8.6% and 13.5%, respectively, while the SAA concentrations were also measured using a commercial Human SAA ELISA Kit (FineTest Biotech Inc., Wuhan, Hubei, China), with intra-assay and inter-assay coefficients of variance equal to 6.4% and 9.9%, respectively.

S1P was quantified using the HPLC-MS/MS method using HPLC-grade analytical standards (Avanti Polar Lipids, Birmingham, AL, USA). A liquid–liquid extraction method was used for lipid extraction from the plasma samples. A total of 30 µL of HPLC-grade methanol was used to dry and reconstitute the final extracts. Sphingolipids were chromatographically separated using a Zorbax Eclipse Plus C8 column (4.6 × 150 mm, 5 m) from Agilent Technologies. Quantification was performed using the Agilent 6420 triple quad with an ESI ion source in MRM data acquisition mode (Agilent Technologies, Santa Clara, CA, USA). The source conditions were gas and vaporizer temperatures of 340 °C and 250 °C, respectively; a nebulizer pressure of 20 psi; a gas flow rate of 12 L/min; a positive capillary voltage of 4500 V; and a negative capillary voltage of 4000 V.

### 2.3. Statistical Analysis

The distribution of the data was tested using the Kolmogorov–Smirnov and Shapiro–Wilk tests. The data are presented as arithmetic means and standard deviation for normally distributed parameters; geometric means and 95th confidence intervals for log-normal values; and medians and interquartile range for parameters which were non-normally distributed even after logarithmic transformation. Categorical variables are shown as relative or absolute frequencies. Continuous variables normally and log-normally distributed as a function of time were compared using repeated-measures analysis of variance (ANOVA), with post hoc Bonferroni correction to reduce the likelihood of chance findings from multiple comparisons, while those that were non-normally distributed were compared using non-parametric repeated-measures ANOVA (Friedman test) with a post hoc Wilcoxon signed-rank test. Differences between normally and log-normally distributed variables were tested using Student’s t-test, while asymmetrically distributed variables were compared using the Mann–Whitney U test. Categorical variables were tested using the Chi-square test. We used univariate and multivariate binary logistic regression to seek possible associations between the parameters investigated and the development of pregnancy complications. The control group was defined as the reference group and coded as 0, while the group with complications was defined as the outcome group and coded as 1. The univariate analysis distinguished individual parameters associated with pregnancy complication development, while multivariate analysis demarcated parameters which were associated with pregnancy complication development independently of the influence of other significant predictors. Statistical tests were considered significant at the 0.05 probability level. The statistics package PASW Statistics 18 (IBM, Armonk, NY, USA) was used for all the statistical analyses.

## 3. Results

The general characteristics of the groups of pregnant women studied are presented in Table 1. The pregnant women in the control group and the group with complications were matched by age and did not significantly differ by smoking status before pregnancy. The women that experienced complications had significantly higher pregestational BMIs, while no significant differences in pregnancy weight gain between the examined groups were identified (Table 1). Further, a significantly higher percentage of pregnant women had used pregestational vitamin supplementation in the group that experienced complications.

Table 2 outlines the longitudinal changes in the MAP, glucose concentration, body weight, BMI, and routine lipid profile parameters in both groups of pregnant women. The MAP was significantly higher in the second and third trimesters than in the first trimester for the control group (*p* < 0.05). In the group with complications, their MAP was significantly lower in the second and third trimesters compared with the first trimester (*p* < 0.05). A significant decrease in glucose concentrations in the second and third trimester was also found for the control group (*p* < 0.05), while there were no differences in the group with complications (*p* = 0.158). Body weight and BMI significantly increased during pregnancy in both groups investigated (*p* < 0.001), as did their TC, TG, and LDL-C concentrations across all three trimesters of pregnancy, which was expected. The HDL-C concentrations showed a significant increase in the second and third trimesters compared with the first (*p* < 0.05) in the control group, while in the group that experienced complications, we noticed a slight but not statistically significant increase in their HDL-C concentrations in the second trimester compared with the first.

In the first trimester, MAP, body weight, BMI, and TG concentrations were significantly higher in the group with complications than in the control group. In the second trimester, MAP, body weight, BMI, and glucose and TG concentrations were significantly higher in the group with complications, while in the third trimester, their TC and LDL-C concentrations were significantly lower (Table 2). Body weight and BMI were significantly higher in the group with complications (*p* < 0.001) in the third trimester. No significant differences in HDL-C concentrations throughout pregnancy were found between the groups investigated (Table 2).

Table 3 shows data for both groups investigated on the trajectory of changes in their oxidative stress status and inflammatory parameters throughout pregnancy. We did not identify any significant changes in the TAC, TOS, or PAB concentrations through pregnancy in the control group (Table 3). The TAC and PAB concentrations were significantly higher in the second trimester than in the first trimester for the group that experienced complications (*p* < 0.05). The TAC concentrations were significantly lower in the group with complications than the control group throughout the entirety of pregnancy, while their TOS concentrations were significantly higher (Table 3). No significant difference in PAB values was found in the first trimester, but during the second and third trimesters, these values were significantly higher in the group with complications (Table 3). In the group that experienced complications, their hsCRP concentrations were significantly higher in the second trimester than in the first trimester (*p* < 0.05), but we did not find any other significant changes throughout pregnancy in this parameter in either of the groups (Table 3). MCP-1 concentrations significantly decreased over the course of pregnancy in both groups investigated. In the first trimester, the MCP-1 concentrations were significantly higher in the group with complications (*p* < 0.05). No significant differences in the inflammatory markers investigated were found between the control group and the group with complications in the second trimester, but in the third trimester, the group with complications had significantly higher MCP-1 concentrations (Table 3).

Changes in the HDL-associated antioxidative and inflammatory markers in the study groups across the trimesters of pregnancy are presented in Table 4. The results obtained show a significant increase in the S1P concentrations in the third trimester compared with the first for the control group. A similar, albeit swifter, trend was found in the group with complications, since this significant increase was observed in the second trimester (Table 4). In both groups investigated, a significant decrease was found in their Apo M concentrations in the second and third trimesters compared with the first trimester (Table 4). The S1P/Apo M ratio significantly increased in the second trimester in both groups investigated and remained significantly higher in the third trimester compared with that at the beginning of pregnancy (Table 4). Apo A1 concentrations showed a significant increase in the second and third trimesters compared with the first in both groups investigated (*p* < 0.001). In the control group, their PON1 concentrations significantly increased in the second trimester (*p* < 0.001) and then noticeably decreased in the third trimester (*p* < 0.05), with the same change trend recorded for the group with complications. Meanwhile, their PON1 activity significantly decreased in the second trimester compared with the first trimester and then significantly increased in the third trimester compared with the second trimester, while there were no changes in PON1 activity across trimesters in the group with complications (*p* = 0.856). The SAA levels significantly increased across trimesters in the control group (*p* < 0.001), while there were no differences in concentrations in the group with complications (*p* = 0.171). No significant differences in Apo A1 concentrations were recorded between the control group and the group with complications in the first trimester, but in the second and third trimesters, the Apo A1 concentrations were significantly higher in the group with complications (*p* < 0.001, *p* < 0.05, respectively). The S1P, PON1, and SAA concentrations were significantly lower in the group with complications in the first trimester (Table 4). During the second trimester, only their S1P/Apo M ratio and SAA concentrations were significantly lower, but in the third trimester, their PON1, apolipoprotein M (Apo M), and SAA concentrations were all significantly lower.

We tested the ability of the parameters investigated to predict the development of pregnancy complications using univariate binary logistic regression analysis (Table 5), with the results emphasizing age, pregestational BMI, MAP, pregestational vitamin supplementation, TGs, TAC, TOS, SAA, glucose, PON1, and S1P levels as potential markers which could identify the group that experienced complications.

Subsequently, we developed a logistic regression model to test for the potential independent association of the parameters investigated with the onset of cardiometabolic pregnancy complications. The model incorporated all predictors of significance (*p* < 0.1) from the univariate analysis (Table 6), with the exception of TOS in order to avoid multicollinearity with TAC. The observed predictive capacities of age, MAP, and glucose, TG, SAA, and PON1 concentrations were lost after accounting for the associated effects of all significant predictors; however, the association of a higher pregestational BMI, a lower TAC, lower S1P levels, and pregestational vitamin supplementation with pregnancy complications remained strong irrespective of the other variables. Therefore, these parameters show a significant independent association with the development of pregnancy complications (Nagelkerke R Square 0.592).

## 4. Discussion

In this study, we sought to identify the possible differences in HDL remodeling between healthy pregnancies and pregnancies with maternal cardiometabolic complications, gestational diabetes, and hypertensive pregnancy disorders. According to our results, during pregnancies with maternal cardiometabolic complications, more intensive oxidative stress, weakened antioxidant defense, and a greater extent of inflammation precipitate an unfavorable microenvironment and affect HDL composition and functionality. Furthermore, in connection with HDL, we identified the S1P levels in the initial trimester as a marker independently associated with the development of cardiometabolic complications.

Comprehensive approaches to developing our molecular understanding of HDL, including proteomic and lipidomic analyses, have shown that the composition of HDL is highly diverse and complex. These extensive insights have implicated HDL and ultimately proven its involvement in multiple antiatherogenic functions, including reverse cholesterol transport and anti-inflammatory, antioxidative, vasodilatory, and anti-thrombotic actions [31]. Given that HDL is crucial to maintaining cardiometabolic health, evidently, the proper metabolism and functionality of this lipoprotein may be key in preventing maternal cardiometabolic complications during pregnancy [32].

In this study, the pregnant women in the group with complications had significantly higher pregestational BMIs compared with the control group, while they did not differ by pregnancy weight gain (Table 1). However, during the entirety of pregnancy, body weight and BMI were statistically significantly higher in the group that experienced complications (Table 2). Although women who were obese pre-pregnancy were excluded from this study, this observation shows that even being overweight pregestationally can increase the likelihood of metabolic complications during pregnancy, highlighting the importance of maternal metabolic status pre-pregnancy to favorable pregnancy outcomes. In this study, we confirmed the expected trajectory for routine lipid parameters (Table 2). At the early stage of pregnancy, during the first trimester, only the TG concentrations were significantly higher in the group with complications than in the control group (Table 2). This finding confirms the established link between hypertriglyceridemia at the start of pregnancy and the risk of developing metabolic pregnancy complications [33,34]. It is noteworthy that no significant differences in the HDL-C concentrations between the two groups were found throughout the entire course of pregnancy; however, in the control group, their HDL-C concentrations significantly increased in the second trimester and remained higher until birth, while this trend of change was not observed in the group that experienced complications (Table 2). A growing body of evidence suggests that an increase in HDL-C levels is expected in healthy pregnant women [35,36], while no such change may be associated with pregnancy complications, especially gestational diabetes and preeclampsia [37,38].

Our study results indicate more intensive oxidative stress and a higher level of inflammation in the group with complications (Table 3). Oxidative stress is a significant regulator of metabolic homeostasis, impacting female fertility, pregnancy, and pregnancy outcomes [39]. We found significantly lower TAC, higher TOS, and higher PAB concentrations for the entire course of pregnancy in the group that experienced complications (Table 3). Inflammatory status during physiological pregnancy is unique and complex, constituting a pro-inflammatory environment during placental development, followed by an anti-inflammatory milieu to ensure maternal tolerance of the fetus [40]. In this study, we have demonstrated significant differences in the MCP-1 concentrations in the first and third trimesters between the two groups investigated (Table 3). Previous research has also suggested that MCP-1 may influence immune response, angiogenesis, and pregnancy outcomes, indicating that higher concentrations might be associated with the development of pregnancy complications [41]. Both groups we studied showed a trend of a slight increase in their hsCRP concentrations during pregnancy, but we only found significantly higher concentrations in the group with complications in the second trimester (Table 3). hsCRP concentrations are widely known to be higher in gestational diabetes and hypertensive pregnancy disorders. Additionally, some authors have proposed potentially screening for this biomarker to predict pregnancy complications [42].

In our research, beyond recording the HDL-C concentrations, we analyzed the trajectory of changes in HDL components that may impact its functionality throughout healthy pregnancy and pregnancy with cardiometabolic complications. We uncovered a changed and unfavorable microenvironment with more intensive oxidative stress and higher levels of inflammation in the latter case. Indeed, the atheroprotective biological effects of HDL, such as vasodilation, antioxidative potential, and anti-inflammatory functions, are impacted by S1P [43]. For the past decade, circulating S1P has been studied extensively as a biomarker involved in the pathogenesis of cardiometabolic diseases and S1P signaling disorders, with an unfavorable S1P metabolism reported as a common feature of several cardiometabolic diseases [44]. In this study, we found a significant increase in the S1P concentrations as pregnancy progressed in both groups investigated (Table 4). Dobierzewska et al. [45] recorded no significant changes in the plasma levels of S1P during healthy gestation or in the context of preeclampsia. In contrast, Melland-Smith et al. [46] revealed a reduction in S1P concentrations in preeclampsia in the third trimester. Since data regarding the longitudinal trajectory of S1P during pregnancy is absent in the literature, our results may imply that an increase in S1P plasma levels toward the end of pregnancy could be an adaptive HDL-related mechanism involved in preserving maternal vascular function during pregnancy. S1P concentrations have previously been recorded as unchanged, higher, and significantly lower in preeclampsia and gestational diabetes compared with uncomplicated pregnancy [45,46,47]. We found that the S1P concentrations were significantly lower in the first trimester in the group with complications than in the control group, while we found no significant differences in the S1P concentrations during the second and third trimesters (Table 4). According to our results, a lower S1P-mediated HDL vascular protective function at the beginning of pregnancy could presumably be associated with the development of complications.

Although we know that plasma Apo M is mainly bound to HDL, linking HDL and S1P and enabling S1P’s biological function, Apo M’s complete physiological role in relation to HDL requires further clarification. It has also been suggested that Apo M facilitates the transformation of preβ-HDL into mature HDL particles and that lower plasma Apo M concentrations are connected with cardiometabolic and inflammatory diseases [48]. Our study results showed a decrease in Apo M concentrations in the second trimester in both groups investigated (Table 4), as well as significantly lower Apo M concentrations in the group with complications in the third trimester than the control group (Table 4). These results suggest that Apo M, as well as the S1P content of HDL, differs in pregnancy with cardiometabolic complications and may lead to deteriorated HDL function. Previous research by Jiang et al. [49] came to similar conclusions in that they also noticed a significant decrease in Apo M concentrations during the second and third trimesters compared with the beginning of pregnancy and lower Apo M concentrations in gestational diabetes. On the contrary, Ahnstrom et al. [50] noted an increase in Apo M concentrations during pregnancy and found no differences between healthy pregnancy and various pregnancy complications. The relatively small sample sizes in the studies mentioned explain the discrepancies in the results obtained, suggesting the need for further investigations. The synergistic effect of S1P and APO M in explaining HDL functionality may be complex. Our study results showed that the S1P/Apo M ratio increased in the second trimester in both groups investigated, reflecting the importance of these parameters to HDL-mediated vascular protection during pregnancy (Table 4). It is of particular interest that this ratio was significantly lower in the second trimester for the group that experienced complications, indicating that HDL-mediated protection of the endothelium may be implicated in the development of cardiometabolic complications during pregnancy. Previous study results have also highlighted a higher S1P concentration and consequently a higher S1P/Apo M ratio as potential markers for maternal adaptation to pregnancy [17], but further research is necessary to enhance the existing evidence.

Our results showed the expected pattern of changes in the Apo A1 concentrations throughout pregnancy in both groups investigated, which manifested as a significant increase in the second trimester (Table 4). The biological mechanism accounting for increased Apo A1 in pregnancy is higher estrogen-induced synthesis in the liver. Recent investigations further explaining this metabolic alteration in pregnancy are very intriguing, suggesting that the placenta has bi-directional potential to secrete apoA1, with a predominant orientation toward the maternal side, and direct effects on maternal lipid metabolism and cholesterol homeostasis [51]. Although Apo A1 has a confirmed atheroprotective role and positive effect on cardiometabolic status in the general population, its implications for cardiometabolic pregnancy complications are still the subject of scientific debate. Previous research findings related to Apo A1 concentrations in cardiometabolic pregnancy complications, gestational diabetes, and preeclampsia are inconsistent [20,52,53]. Our results showed higher Apo A1 concentrations towards the end of pregnancy in the group that experienced complications; this could be explained by growing evidence that placental overexpression of the Apo A1 gene is linked to negative pregnancy outcomes [51,53]. In pregnancy complications, an altered, unfavorable maternal environment during pregnancy, including oxidative stress, inflammation, hyperglycemia, and hyperlipidemia, may increase placental Apo A1 production. In turn, all these factors lead to molecular alterations in ApoA1, modifying its antiatherogenic capacity and therefore HDL functionality [54]. However, the mechanism behind the paradoxical association of high levels of Apo A1 with cardiometabolic pregnancy complications is still indefinite.

PON1 is a crucial component of the HDL proteome and its antioxidative capacity [55]. Our study results showed the same trend of changes in the PON1 concentrations during pregnancy in both groups investigated (Table 4), with significant increases in the second trimester and then significant decreases in the third trimester. We also found significantly lower PON1 concentrations in the group with complications in the first and third trimesters. Adaptive structural metabolic changes in HDL due to higher demands for antioxidants could explain the increase in HDL PON1 content mid-gestation. However, this increase in the PON1 concentrations throughout pregnancy was not accompanied by an equivalent increase in PON1 activity (Table 4). We found that PON1 activity significantly decreased in the second trimester in the control group and then increased in the third trimester, while the PON1 activity was unchanged during pregnancies with complications (Table 4). Our results in the control group are in agreement with previous research [56], with the observed decrease in the second trimester attributed to the exhaustion of HDL’s antioxidative capacity in a microenvironment with intense oxidative stress. Further, the decrease in HDL PON1 content during the second trimester implies increased PON1 synthesis and a resultant increase in PON1 activity in the third trimester in healthy pregnancy. Conversely, in the group that experienced complications, the increase in HDL PON1 content over the course of pregnancy did not result in increased PON1 activity (Table 4). It is well established that the oxidation of HDL particles results in decreased PON1 activity [57]; thus, we can assume that in cardiometabolic pregnancy complications, higher levels of inflammation and greater oxidative stress impair PON1 activity, leading to functional changes in HDL particles that affect their antioxidative properties.

SAA is a highly sensitive acute-phase reactant that is primarily associated with circulating HDL particles. It can displace other HDL-associated proteins, such as Apo A1 or PON1, and has the potential to impair HDL functionality [22]. Our study results showed that the SAA concentrations were increased throughout pregnancy in the control group, while no changes were seen for the group that experienced complications (Table 4). Nevertheless, previous research has found that both uncomplicated and complicated pregnancies are accompanied by a significant increase in the SAA levels in maternal circulation [58]. The observed absence of the expected increase in SAA concentrations in the group with complications may be contingent upon placental SAA production during pregnancy greatly impacting the amount of SAA in circulation [59] and this production being affected by the unfavorable microenvironment accompanying cardiometabolic pregnancy complications. Further, for the entirety of pregnancy, the SAA concentrations were significantly lower in the group that experienced cardiometabolic complications (Table 4). While our results are unexpected, the latest research in this field has shifted the previously established perception of how SAA affects HDL functionality. Sato et al. [60] found that SAA affects the composition of HDL particles in displacing apo A1 but that this replacement enhances its antioxidant ability. Jayjamaran et al. [61] discovered that the enrichment of HDL with SAA reduces both HDL and LDL oxidation and that mild oxidation of SAA-enriched HDL results in SAA release, with antioxidant effects. These findings indicate that amending our incomplete understanding of the mediation of SAA in HDL dysfunction could also further elucidate different aspects of HDL’s protective function.

Another focus of interest was our evaluation of the predictive capacity of HDL components in the first trimester for the development of cardiometabolic pregnancy complications. Pregestational BMI, pregestational vitamin supplementation, TAC, and S1P concentrations were significantly and independently associated with cardiometabolic pregnancy complications development regardless of the other variables (Table 6). Our results highlighted S1P in the first trimester as an HDL-associated component that may affect its functionality and potentially predict the development of cardiometabolic pregnancy complications. These findings suggest that the role of S1P in elucidating pathogenesis and risk assessments for cardiometabolic pregnancy complications require further study.

This study had several limitations worth mentioning. The statistical power of the analyses may have been hindered by the small sample size, and some of the observed trends of changes could potentially reach statistical significance in a larger sample. When interpreting the results obtained, the potential role of physiological hemodilution during pregnancy is also a worthy consideration. Another of the limitations of this study is that during the entire pregnancy course, BMI was significantly higher in the group that experienced complications. Since fat tissue can affect the parameters examined, the study’s results should be confirmed in a group of pregnant women with a healthy weight range before pregnancy (18.5 ≤ BMI ≤ 24.9) who developed cardiometabolic pregnancy complications.

In sum, HDL remodeling follows a different pattern of changes in pregnancy with complications compared to in healthy pregnancy. Considering the crucial bearing of HDL particles on cardiometabolic status, it is possible that divergence from the expected HDL metabolism associated with healthy pregnancy may affect HDL functionality during pregnancies with cardiometabolic complications and contribute to unfavorable pregnancy outcomes. According to our findings, components of HDL that determine its antioxidative and anti-inflammatory capacity, especially Apo A1 and SAA content, are altered in cardiometabolic pregnancy complications, which could result in weakened HDL functionality. Our results emphasized the significance of S1P quantification in the first trimester of pregnancy, and we therefore encourage further investigation of it as a reliable biomarker that reflects HDL’s functional capabilities. With increasing evidence of the importance of lipid metabolism during pregnancy to the health of both mother and child, determining routine lipid profile parameters in pregnant women through laboratory evaluation is subject to considerable scientific and clinical debate. In the context of the current study, measuring HDL-C in the second trimester might lead to more comprehensive risk assessments, earlier and more frequently assessing pregnant women at risk, and ultimately improve pregnancy outcomes. However, precise definition of the cutoff values suggestive of cardiometabolic pregnancy complications is necessary to fully implement HDL-C in everyday laboratory practice.

## Figures and Tables

**Table 1:** General characteristics of study groups.

	Control Group	Group with Complications	*p*
N	84	46	
Age (years)	31.6 ± 5.42	33.3 ± 5.46	0.081
Pregestational BMI (kg/m^2^)	21.7 ± 2.89	23.9 ± 3.63	<0.001
Smoking before pregnancy (%) ^#^	27.4	28.3	0.551
Pregestational vitamin supplementation (%) ^#^	35.7	71.7	<0.001
Positive family history for cardiometabolic diseases (%) ^#^	58.3	52.1	0.486
First pregnancy (%) ^#^	40.4	36.7	0.721
Polycystic ovary syndrome before pregnancy (N)	-	3	
Hashimoto’s thyroiditis before pregnancy (N)	-	4	
Pregnancy weight gain (kg) *	17.5 (11.5–22.5)	15.0 (9.5–2.5)	0.092
Gestational diabetes (N)	-	13	
Gestational hypertension (N)	-	15	
Preeclampsia (N)	-	12	
Gestational diabetes and gestational hypertension (N)	-	2	
Gestational diabetes and preeclampsia (N)	-	4	

Data are presented as means ± standard deviation and compared using Student’s t-test. ^#^ Data are presented as relative frequencies and compared using the Chi-square test. * Data are presented as medians (interquartile ranges) and compared using the Mann–Whitney U-test.

**Table 2:** Changes in MAP, glucose, body weight, BMI, and general lipid profile parameter concentrations in study groups across trimesters of pregnancy.

	1st Trimester			2nd Trimester			3rd Trimester			*P*_1_	*P*_2_
	Control Group (N = 84)	Group with Complications (N = 46)	*p*	Control Group (N = 84)	Group with Complications (N = 46)	*p*	Control Group (N = 84)	Group with Complications (N = 46)	*p*		
>MAP ^&^	87.3 (80.0–93.3)	93.7 (86.0–102.2)	<0.001	82.5 ^a^* (73.3–88.3)	89.5 ^a#^ (82.9–89.5)	<0.001	83.3 ^a^* (78.5–87.7)	86.3 ^a#^ (78.6–93.3)	0.090	<0.001	<0.05
>Glucose, mmol/L	4.6 ± 0.40	4.8 ± 0.75	0.054	4.4 ± 0.47 ^a#^	4.7 ± 0.70	<0.05	4.5 ± 0.46 ^a#^	4.6 ± 0.6	0.173	<0.05	0.158
>Body weight, kg	63.2 ± 8.95	70.9 ± 12.98	<0.001	67.1 ± 9.21 ^a^*	76.2 ± 13 ^a^*	<0.001	70.7 ± 11.79 ^a^*^, b^*	81.7 ± 13.76 ^a^*^, b^*	<0.001	<0.001	<0.001
>BMI, kg/m ^2&^	22.0 (20.0–23.5)	25.0 (22.1–28.9)	<0.001	23.4 ^a^* (21.5–24.9)	26.7 ^a^* (24.4–29.5)	<0.001	24.5 ^a^*^, b^* (23.1–26.7)	28.5 ^a^*^, b^* (26.8–32.0)	<0.001	<0.001	<0.001
>TC, mmol/L	5.3 ± 0.97	5.3 ± 0.94	0.887	6.7 ± 1.34 ^a^*	6.6 ± 1.39 ^a^*	0.474	7.2 ± 1.46 ^a^*^,b^*	6.6 ± 1.34 ^a^*	<0.05	<0.001	<0.001
>TG, mmol/L ^†^	1.23 (1.13–1.35)	1.51 (1.32–1.72)	<0.05	1.82 ^a^* (1.66–1.99)	2.10 ^a^* (1.86–2.36)	<0.05	2.28 ^a^*^,b^* (2.06–2.52)	2.60 ^a^*^,b^* (2.35–2.88)	0.258	<0.001	<0.001
>HDL-C, mmol/L	1.7 ± 0.31	1.6 ± 0.35	0.589	1.9 ± 0.32 ^a^*	1.8 ± 0.39	0.280	1.8 ± 0.31 ^b^*	1.7 ± 0.33	0.736	<0.05	0.479
>LDL-C, mmol/L	3.0 ± 0.86	2.8 ± 0.81	0.185	4.1 ± 1.21 ^a^*	3.7 ± 1.17 ^a^*	0.117	4.2 ± 1.22 ^a^*^,b#^	3.6 ± 1.13 ^a^*	<0.05	<0.05	<0.001

Data are presented as means ± standard deviation. ^†^ Data are presented as geometric means (95th CIs). *p*—Student’s *t*-test for between-group differences; *p*_1_—repeated-measures ANOVA for control group; *p*_2_—repeated-measures ANOVA for group with complications. Pairwise comparison: ^a^ mean difference significantly different from the first trimester; ^b^ mean difference significantly different from the second trimester; * *p* < 0.001 (Bonferroni-corrected); ^#^ *p* < 0.05 (Bonferroni-corrected). ^&^ Data are presented as medians (interquartile ranges). *p*—Mann–Whitney U-test for between-group differences. *p*_1_—Friedman test (non-parametric repeated-measures ANOVA) for control group; *p*_2_—Friedman test (non-parametric repeated-measures ANOVA) for group with complications. Pairwise comparison: ^a^ significantly different from the first trimester; ^b^ significantly different from the second trimester; * *p* < 0.001 (Wilcoxon signed-rank test); ^#^ *p* < 0.05 (Wilcoxon signed-rank test).

**Table 3:** Changes in oxidative stress status and inflammatory parameters in study groups across trimesters of pregnancy.

	1st Trimester			2nd Trimester			3rd Trimester			*P*_1_	*P*_2_
	Control Group (N = 84)	Group with Complications (N = 46)	*p*	Control Group (N = 84)	Group with Complications (N = 46)	*p*	Control Group (N = 84)	Group with Complications (N = 46)	*p*		
TAC, mmol/L	1.063 (0.992–1.131)	0.756 (0.629–1.064)	<0.001	1.102 (0.973–1.213)	0.91 ^a#^ (0.739–1.075)	<0.001	1.029 (0.877–1.171)	0.859 (0.772–1.054)	<0.001	0.101	<0.05
TOS, μmol/L	7.7 (6.42–9.17)	11.6 (7.70–17.46)	<0.001	6.7 (5.7–10.8)	10.0 (6.6–17.6)	<0.05	8.0 (6.6–9.7)	9.4 (6.6–14.7)	0.059	0.141	0.899
PAB, HK units	194.9 (162.72–215.96)	176.7 (150.80–230.22)	0.608	157.57 (142.44–190.33)	190.63 ^a#^ (154.41–257.40)	<0.001	169.8 (149.9–187.48)	187.62 (165.25–228.20)	<0.001	0.707	<0.05
hsCRP, mg/L	3.90 (2.32–7.12)	3.85 (2.42–6.40) 1.72)	0.806	4.40 (2.30–8.47)	4.60 ^a#^ (2.37–7.92)	0.979	4.10 (2.9–6.9)	4.15 (1.97–8.87)	0.886	0.176	<0.05
MCP-1, pg/ml	162.56 (106.01–199.80)	201.70 (133.03–325.47)	<0.05	129.5 ^a^* (89.31–166.54)	139.97 ^a^* (99.73–199.96)	0.313	70.78 ^a^* (48.03–100.40)	142.90 ^a#^ (71.92–222.47)	<0.001	<0.001	<0.001

Data are presented as medians (interquartile ranges). *p*—Mann–Whitney U-test for between-group differences. *p*_1_—Friedman test (non-parametric repeated-measures ANOVA) for control group; *p*_2_—Friedman test (non-parametric repeated-measures ANOVA) for group with complications; pairwise comparison: ^a^ significantly different from the first trimester; * *p* < 0.001 (Wilcoxon signed-rank test); ^#^ *p* < 0.05 (Wilcoxon signed-rank test).

**Table 4:** Changes in HDL-associated antioxidative and inflammatory markers in study groups across trimesters of pregnancy.

	1st Trimester			2nd Trimester			3rd Trimester			*P*_1_	*P*_2_
	Control Group (N = 84)	Group with Complications (N = 46)	*p*	Control Group (N = 84)	Group with Complications (N = 46)	*p*	Control Group (N = 84)	Group with Complications (N = 46)	*p*		
S1P, nmol/L ^†^	477.8 (337.8–980.7)	432.2 (349.6–601.1)	<0.05	496.9 (377.4–749.8)	532.7 ^a#^ (404.8–701.9)	0.881	557.5 ^a#^ (405.1–833.4)	537.1 ^a#^ (410.3–705.6)	0.497	<0.05	<0.05
Apo M, mg/L ^†^	44.9 (25.8–58.9)	44.3 (24.7–63.5)	0.831	25.2 ^a^* (18.9–29.8)	29.8 ^a#^ (23.9–43.8)	0.068	29.7 ^a^* (22.7–39.1)	24.8 ^a#^ (18.1–33.8)	<0.05	<0.001	<0.05
S1P/Apo M ratio	13.7 (6.64–24.08)	12.1 (8.10–14.39)	0.288	22.2 ^a^* (15.57–33.68)	16.3 ^a^* (11.87–21.52)	<0.05	21.1 ^a^* (13.94–32.31)	17.3 ^a^* (13.26–29.63)	0.512	<0.001	<0.001
Apo A1, g/L	1.82 ± 0.273	1.90 ± 0.253	0.098	1.98 ± 0.34 ^a^*	2.20 ± 0.372 ^a^*	<0.001	1.93 ± 0.276 ^a^*	2.10 ± 0.404 ^a^*	<0.05	<0.001	<0.001
PON1, ng/mL ^†^	1259.7 (717.5–1657.2)	1157.1 (868.9–1478.8)	<0.05	1624.3 ^a#^ (1002.3–1832.0)	1655.7 ^a#^ (1266.6–2107.0)	0.501	1168.9 ^b#^ (745.4–1491.3)	1097.9 ^b#^ (738.7–1368.1)	<0.05	<0.05	<0.05
PON1 act., U/L ^†^	311.0 (235.0–806.2)	318.0 (246.5–669.0)	0.667	287.0 ^a#^ (228.2–727.7)	351.0 (264.5–652.5)	0.432	305.5 ^b#^ (246.0–726.5)	393.0 (229.0–779.5)	0.998	<0.05	0.856
SAA, ng/L ^†^	86.9 (67.2–112.2)	50.3 (23.3–79.1)	<0.001	101.5 ^a#^ (77.6–147.1)	47.5 (19.3–88.7)	<0.001	150.4 ^a^*^,b#^ (117.4–188.9)	50.8 (12.6–138.6)	<0.001	<0.001	0.171

Data are presented as means ± standard deviation and compared using Student’s t-test and repeated-measures ANOVA. Pairwise comparison: ^a^ mean difference significantly different from the first trimester; ^b^ mean difference significantly different from the second trimester; * *p* < 0.001 (Bonferroni-corrected); ^#^ *p* < 0.05 (Bonferroni-corrected). ^†^ Data are presented as medians (interquartile ranges). *p*—Mann–Whitney U-test for between-group differences. *p*_1_—Friedman test (non-parametric repeated-measures ANOVA) for control group; *p*_2_—Friedman test (non-parametric repeated-measures ANOVA) for group with complications; pairwise comparison: ^a^ significantly different from the first trimester; ^b^ significantly different from the second trimester; * *p* < 0.001 (Wilcoxon signed-rank test); ^#^ *p* < 0.05 (Wilcoxon signed-rank test).

**Table 5:** Univariate binary logistic regression analysis for associations between the examined parameters and pregnancy complication development.

Parameter	OR	95%CI	*p*
>Age, years	1.062	0.992–1.136	0.083
>BMI, kg/m^2^	1.238	1.093–1.402	<0.05
>MAP	1.068	1.026–1.113	<0.05
>Pregestational prenatal vitamin supplementation	4.569	2.091–9.985	<0.001
>Glucose, mmol/L	1.916	0.947–3.878	0.071
>TC, mmol/L	0.973	0.669–1.416	0.886
>TGs, mmol/L	2.265	1.044–4.965	<0.05
>HDL-C, mmol/L	0.730	0.236–2.261	0.586
>LDL-C, mmol/L	0.735	0.466–1.161	0.187
>TAC, mmol/L	0.994	0.992–0.997	<0.001
>TOS, μmol/L	1.139	1.061–1.222	<0.001
>PAB, HK unites	0.999	0.99–1.099	0.896
>hsCRP, mg/L	0.985	0.922–1.053	0.663
>MCP-1, pg/ml	1.002	0.999–1.004	0.166
>PON1, ng/ml	0.999	0.999–1.000	0.096
>PON1 act, U/L	1.000	0.999–1.001	0.419
>Apo A1, g/L	0.992	0.936–1.098	0.552
>Apo M, mg/L	0.996	0.979–1.014	0.655
>SAA, ng/L	0.981	0.969–0.992	<0.05
>S1P, nmol/L	0.999	0.998–1.000	0.073

**Table 6:** Multivariate binary regression analysis for independent associations between significant parameters for pregnancy complication development.

Parameter	OR	95%CI	*p*
>Age, years	1.070	0.969–1.181	0.181
>BMI, kg/m^2^	1.292	1.078–1.549	<0.05
>MAP	1.053	1.000–1.109	0.051
>Glucose, mmol/L	1.893	0.664–5.396	0.232
>TGs, mmol/L	2.634	0.852–8.143	0.098
>SAA, ng/L	0.995	0.984–1.007	0.426
>TAC, mmol/L	0.994	0.990–0.997	<0.001
>PON1, ng/ml	1.000	0.998–1.001	0.799
>S1P, nmol/L	0.998	0.997–1.000	<0.05
>Pregestational prenatal vitamin supplementation	3.871	1.144–13.103	<0.05
>Model Summary: Nagelkerke R Square 0.592			

## Data Availability

The data presented in this study are available on request from the corresponding author. The data are not publicly available due to privacy and ethics considerations.

## References

[1] Parrettini S., Caroli A., Torlone E. (2020). Nutrition and Metabolic Adaptations in Physiological and Complicated Pregnancy: Focus on Obesity and Gestational Diabetes. Front. Endocrinol..

[2] Mulder J.W., Kusters D.M., van Lennep J.E.R., Hutten B.A. (2024). Lipid Metabolism during Pregnancy: Consequences for Mother and Child. Curr. Opin. Lipidol..

[3] Abu-Awwad S.-A., Craina M., Boscu L., Bernad E., Ciordas P.D., Marian C., Iurciuc M., Abu-Awwad A., Iurciuc S., Bernad B. (2023). Lipid Profile Variations in Pregnancies with and without Cardiovascular Risk: Consequences for Both Mother and Newborn. Children.

[4] Jiang L., Tang K., Magee L.A., von Dadelszen P., Ekeroma A., Li X., Zhang E., Bhutta Z.A. (2022). A Global View of Hypertensive Disorders and Diabetes Mellitus during Pregnancy. Nat. Rev. Endocrinol..

[5] Ardalić D., Stefanović A., Banjac G., Cabunac P., Miljković M., Mandić-Marković V., Stanimirović S., Pažin B.D., Spasić S., Spasojević-Kalimanovska V. (2020). Lipid Profile and Lipid Oxidative Modification Parameters in the First Trimester of High- Risk Pregnancies—Possibilities for Preeclampsia Prediction. Clin. Biochem..

[6] Antonić T.D., Ardalić D., Vladimirov S.S., Banjac G.S., Cabunac P.J., Zeljković A.R., Karadžov-Orlić N.T., Spasojević-Kalimanovska V.V., Miković .D., Stefanović A. (2021). Cholesterol Homeostasis Is Dysregulated in Women with Preeclampsia. Pol. Arch. Med. Wewn.-Pol. Arch. Intern. Med..

[7] Zeljković A., Ardalić D., Vekić J., Antonić T., Vladimirov S., Rizzo M., Gojković T., Ivanišević J., Mihajlović M., Vujčić S. (2022). Effects of Gestational Diabetes Mellitus on Cholesterol Metabolism in Women with High-Risk Pregnancies: Possible Implications for Neonatal Outcome. Metabolites.

[8] Wang H., Dang Q., Zhu H., Liang N., Le Z., Huang D., Xiao R., Yu H. (2020). Associations between Maternal Serum HDL-c Concentrations during Pregnancy and Neonatal Birth Weight: A Population-Based Cohort Study. Lipids Health Dis..

[9] Spracklen C.N., Smith C.J., Saftlas A.F., Robinson J.G., Ryckman K.K. (2014). Maternal Hyperlipidemia and the Risk of Preeclampsia: A Meta-Analysis. Am. J. Epidemiol..

[10] Woollett L.A., Catov J.M., Jones H.N. (2022). Roles of Maternal HDL during Pregnancy. Biochim. Biophys. Acta Mol. Cell Biol. Lipids.

[11] Gallo L.A., Barrett H.L., Nitert M.D. (2017). Review: Placental Transport and Metabolism of Energy Substrates in Maternal Obesity and Diabetes. Placenta.

[12] Jomard A., Osto E. (2020). High Density Lipoproteins: Metabolism, Function, and Therapeutic Potential. Front. Cardiovasc. Med..

[13] Bonizzi A., Piuri G., Corsi F., Cazzola R., Mazzucchelli S. (2021). HDL Dysfunctionality: Clinical Relevance of Quality rather than Quantity. Biomedicines.

[14] Cao P., Pan H., Xiao T., Zhou T., Guo J., Su Z. (2015). Advances in the Study of the Antiatherogenic Function and Novel Therapies for HDL. Int. J. Mol. Sci..

[15] Phoswa W.N., Khaliq O.P. (2021). The Role of Oxidative Stress in Hypertensive Disorders of Pregnancy (Preeclampsia, Gestational Hypertension) and Metabolic Disorder of Pregnancy (Gestational Diabetes Mellitus). Oxidative Med. Cell. Longev..

[16] Kerage D., Gombos R.B., Wang S., Brown M., Hemmings D.G. (2021). Sphingosine 1-Phosphate-Induced Nitric Oxide Production Simultaneously Controls Endothelial Barrier Function and Vascular Tone in Resistance Arteries. Vasc. Pharmacol..

[17] Patanapirunhakit P., Karlsson H., Mulder M., Ljunggren S., Graham D., Freeman D. (2021). Sphingolipids in HDL—Potential Markers for Adaptation to Pregnancy?. Biochim. Biophys. Acta Mol. Cell Biol. Lipids.

[18] Del Gaudio I., Sreckovic I., Zardoya-Laguardia P., Bernhart E., Christoffersen C., Frank S., Marsche G., Illanes S.E., Wadsack C. (2020). Circulating Cord Blood HDL-S1P Complex Preserves the Integrity of the Feto-Placental Vasculature. Biochim. Et Biophys. Acta (BBA)-Mol. Cell Biol. Lipids.

[19] Xu X., Song Z., Mao B., Xu G. (2022). Apolipoprotein A1-Related Proteins and Reverse Cholesterol Transport in Antiatherosclerosis Therapy: Recent Progress and Future Perspectives. Cardiovasc. Ther..

[20] Pasternak Y., Biron-Shental T., Ohana M., Einbinder Y., Arbib N., Benchetrit S., Zitman-Gal T. (2020). Gestational Diabetes Type 2: Variation in High-Density Lipoproteins Composition and Function. Int. J. Mol. Sci..

[21] Retnakaran R., Ye C., Connelly P.W., Hanley A.J., Sermer M., Zinman B. (2019). Serum ApoA1 (Apolipoprotein A-1), Insulin Resistance, and the Risk of Gestational Diabetes Mellitus in Human Pregnancy—Brief Report. Arterioscler. Thromb. Vasc. Biol..

[22] Webb N.R. (2021). High-Density Lipoproteins and Serum Amyloid a (SAA). Curr. Atheroscler. Rep..

[23] Garelnabi M., Litvinov D., Mahini H. (2012). Antioxidant and Anti-Inflammatory Role of Paraoxonase 1: Implication in Arteriosclerosis Diseases. N. Am. J. Med. Sci..

[24] National Institute of Health and Excellence (2013). Quality Standard [QS35]: Hypertension in Pregnancy.

[25] Katz E.D., Ruoff B.E., Roberts J., Hedges J. (2004). Commonly Used Formulas and Calculations. Clinical Procedures in Emergency Medicine.

[26] Friedewald W.T., Levy R.I., Fredrickson D.S. (1972). Estimation of the concentration of low-density lipoprotein cholesterol in plasma, without use of the preparative ultracentrifuge. Clin. Chem..

[27] Erel O. (2004). A Novel Automated Direct Measurement Method for Total Antioxidant Capacity Using a New Generation, More Stable ABTS Radical Cation. Clin. Biochem..

[28] Erel O. (2005). A New Automated Colorimetric Method for Measuring Total Oxidant Status. Clin. Biochem..

[29] Alamdari D.H., Paletas K., Pegiou T., Sarigianni M., Befani C., Koliakos G. (2007). A Novel Assay for the Evaluation of the Prooxidant–Antioxidant Balance, before and after Antioxidant Vitamin Administration in Type II Diabetes Patients. Clin. Biochem..

[30] Kotur-Stevuljevic J., Spasic S., Stefanovic A., Zeljkovic A., Bogavac-Stanojevic N., Kalimanovska-Ostric D., Spasojevic-Kalimanovska V., Jelic-Ivanovic Z. (2006). Paraoxonase-1 (PON1) Activity, but Not PON1Q192R Phenotype, Is a Predictor of Coronary Artery Disease in a Middle-Aged Serbian Population. Clin. Chem. Lab. Med. (CCLM).

[31] Endo Y., Fujita M., Ikewaki K. (2023). HDL Functions—Current Status and Future Perspectives. Biomolecules.

[32] Zeljkovic A., Vekic J., Stefanovic A. (2024). Obesity and Dyslipidemia in Early Life: Impact on Cardiometabolic Risk. Metab. Clin. Exp..

[33] Zhu H., He D., Liang N., Lai A., Zeng J., Yu H. (2020). High Serum Triglyceride Levels in the Early First Trimester of Pregnancy Are Associated with Gestational Diabetes Mellitus: A Prospective Cohort Study. J. Diabetes Investig..

[34] Wu D., Zhang J., Xiong Y., Wang H., Lu D., Guo M., Zhang J., Chen L., Fan J., Huang H. (2022). Effect of Maternal Glucose and Triglyceride Levels during Early Pregnancy on Pregnancy Outcomes: A Retrospective Cohort Study. Nutrients.

[35] Saarelainen H., Laitinen T., Raitakari O.T., Juonala M., Heiskanen N., Lyyra-Laitinen T., Viikari J.S., Vanninen E., Heinonen S. (2006). Pregnancy-Related Hyperlipidemia and Endothelial Function in Healthy Women. Circ. J..

[36] Wild R., Feingold K.R. (2000). Effect of Pregnancy on Lipid Metabolism and Lipoprotein Levels. Endotext.

[37] Bao W., Dar S., Zhu Y., Wu J., Rawal S., Li S., Weir N.L., Tsai M.Y., Zhang C. (2017). Plasma Concentrations of Lipids during Pregnancy and the Risk of Gestational Diabetes Mellitus: A Longitudinal Study. J. Diabetes.

[38] Stadler J.T., Scharnagl H., Wadsack C., Marsche G. (2023). Preeclampsia Affects Lipid Metabolism and HDL Function in Mothers and Their Offspring. Antioxidants.

[39] Hussain T., Murtaza G., Metwally E., Kalhoro D.H., Kalhoro M.S., Rahu B.A., Sahito R.G.A., Yin Y., Yang H., Chughtai M.I. (2021). The Role of Oxidative Stress and Antioxidant Balance in Pregnancy. Mediat. Inflamm..

[40] Weng J., Couture C., Girard S. (2023). Innate and Adaptive Immune Systems in Physiological and Pathological Pregnancy. Biology.

[41] Bogavac M.A., Ćelić D.D., Perić T.M. (2022). A Prospective Study of Mid-Trimester MCP-1 Levels as a Predictor of Preterm Delivery. Medicines.

[42] Kumari R., Singh H. (2017). The Prevalence of Elevated High-Sensitivity C-Reactive Protein in Normal Pregnancy and Gestational Diabetes Mellitus. J. Fam. Med. Prim. Care.

[43] Diarte-Añazco E.M.G., Méndez-Lara K.A., Pérez A., Alonso N., Blanco-Vaca F., Julve J. (2019). Novel Insights into the Role of HDL-Associated Sphingosine-1-Phosphate in Cardiometabolic Diseases. Int. J. Mol. Sci..

[44] Borodzicz-Jażdżyk S., Jażdżyk P., Łysik W., Cudnoch-Jȩdrzejewska A., Czarzasta K. (2022). Sphingolipid Metabolism and Signaling in Cardiovascular Diseases. Front. Cardiovasc. Med..

[45] Dobierzewska A., Soman S., Illanes S.E., Morris A.J. (2017). Plasma Cross-Gestational Sphingolipidomic Analyses Reveal Potential First Trimester Biomarkers of Preeclampsia. PLoS ONE.

[46] Melland-Smith M., Ermini L., Chauvin S., Craig-Barnes H., Tagliaferro A., Todros T., Post M., Caniggia I. (2015). Disruption of Sphingolipid Metabolism Augments Ceramide-Induced Autophagy in Preeclampsia. Autophagy.

[47] Johnstone E.D., Westwood M., Dilworth M., Wray J.R., Kendall A.C., Nicolaou A., Myers J.E. (2023). Plasma S1P and Sphingosine Are Not Different prior to Pre-Eclampsia in Women at High Risk of Developing the Disease. J. Lipid Res..

[48] Tydén H., Lood C., Jönsen A., Gullstrand B., Kahn R., Linge P., Kumaraswamy S.B., Dahlbäck B., Bengtsson A.A. (2019). Low Plasma Concentrations of Apolipoprotein M Are Associated with Disease Activity and Endothelial Dysfunction in Systemic Lupus Erythematosus. Arthritis Res. Ther..

[49] Jiang H., Znang J., Zhang Y., Xu N., Xu G. (2014). Changes of serum apolipoprotein M level in women during normal and gestational diabetes mellitus. Chin. J. Endocrinol. Metabolism..

[50] Ahnström J., Lindqvist P.G., Walle U., Dahlbäck B. (2010). Plasma Levels of Apolipoprotein M in Normal and Complicated Pregnancy. Acta Obstet. Et Gynecol. Scand..

[51] Melhem H., Kallol S., Huang X., Lüthi M., Ontsouka C.E., Keogh A., Stroka D., Thormann W., Schneider H., Albrecht C. (2019). Placental Secretion of Apolipoprotein A1 and E: The Anti-Atherogenic Impact of the Placenta. Sci. Rep..

[52] Gil-Acevedo L., Ceballos G., Torres-Ramos Y. (2022). Foetal Lipoprotein Oxidation and Preeclampsia. Lipids Health Dis..

[53] Liu Z., Pei J., Zhang X., Wang C., Tang Y., Liu H., Yu Y., Luo S., Gu W. (2023). APOA1 Is a Novel Marker for Preeclampsia. Int. J. Mol. Sci..

[54] Faaborg-Andersen C.C., Liu C., Subramaniyam V., Desai S.R., Sun Y.V., Wilson P.W.F., Sperling L.S., Quyyumi A.A. (2022). U-Shaped Relationship between Apolipoprotein A1 Levels and Mortality Risk in Men and Women. Eur. J. Prev. Cardiol..

[55] Denimal D. (2024). Antioxidant and Anti-Inflammatory Functions of High-Density Lipoprotein in Type 1 and Type 2 Diabetes. Antioxidants.

[56] Ferré N., Camps J., Fernández-Ballart J., Arija V., Murphy M.M., Marsillach J., Joven J. (2006). Longitudinal Changes in Serum Paraoxonase-1 Activity throughout Normal Pregnancy. Clin. Chem. Lab. Med. (CCLM).

[57] Kotur-Stevuljević J., Vekić J., Stefanović A., Zeljković A., Ninić A., Ivanišević J., Miljković M., Sopić M., Munjas J., Mihajlović M. (2020). Paraoxonase 1 and Atherosclerosis-Related Diseases. BioFactors.

[58] Lin Y., Zhu P., Wang W., Sun K. (2022). Serum Amyloid A, a Host-Derived DAMP in Pregnancy?. Front. Immunol..

[59] Gan X.-W., Wang W.-S., Lu J.-W., Ling L.-J., Zhou Q., Zhang H.-J., Ying H., Sun K. (2020). De Novo Synthesis of SAA1 in the Placenta Participates in Parturition. Front. Immunol..

[60] Sato M., Ohkawa R., Yoshimoto A., Yano K., Ichimura N., Nishimori M., Okubo S., Yatomi Y., Tozuka M. (2016). Effects of Serum Amyloid a on the Structure and Antioxidant Ability of High-Density Lipoprotein. Biosci. Rep..

[61] Jayaraman S., Haupt C., Gursky O. (2016). Paradoxical Effects of SAA on Lipoprotein Oxidation Suggest a New Antioxidant Function for SAA. J. Lipid Res..

